# Oral Stepdown Therapy for Gram‐Negative Bloodstream Infections: A Systematic Review and Meta‐Analysis

**DOI:** 10.1111/eci.70244

**Published:** 2026-08-13

**Authors:** Lorenzo Onorato, Ilaria De Luca, Marika Ferrante, Giuseppe Ricciardiello, Caterina Monari, Nicola Coppola

**Affiliations:** ^1^ Department of Mental Health and Public Medicine, Section of Infectious Diseases University of Campania Luigi Vanvitelli Naples Italy

**Keywords:** BSI, fluoroquinolones, gram‐negative, oral beta‐lactams, oral treatment

## Abstract

**Background:**

Early oral step‐down has been proposed as a strategy to optimize antimicrobial use in Gram‐negative bloodstream infections (BSIs).

**Methods:**

We performed a systematic review and meta‐analysis of full‐text studies published in English and indexed in MEDLINE and EMBASE up to April 2025. Randomized controlled trials (RCTs) and observational studies including patients with Gram‐negative BSIs managed either with oral step‐down within 5 days of IV therapy or with continued IV therapy were eligible. Risk of bias was assessed using the Cochrane Risk of Bias Tool for RCTs and the Newcastle–Ottawa Scale for observational studies.

**Results:**

Of 4120 screened records, 13 studies (3 RCTs and 10 cohort studies) including 10,000 patients met the inclusion criteria; 5016 of enrolled patients (52%) switched to oral therapy, mainly to fluoroquinolones (2992, 59.6%). A lower all‐cause mortality was observed in patients receiving oral step‐down (RR: 0.39, 95% CI: 0.16–0.93; *p* = 0.034). When stratifying studies according to timing of the switch to oral therapy, no significant difference in mortality was observed in studies with a median switch time ≤ 3 days (*p* = 0.29). Conversely, studies with a median switch time > 3 days showed a lower mortality rate in the oral step‐down group, approaching statistical significance (*p* = 0.051). No significant differences were found between treatment groups in terms of clinical failure (*p* = 0.28). Microbiological failure was significantly less frequent among patients receiving oral step‐down therapy (RR = 0.69, 95% CI: 0.49–0.97; *p* = 0.032), while adverse event rates were comparable (*p* = 0.52). However, sensitivity analyses limited to propensity score–adjusted data demostrated no significant differences in mortality (*p* = 0.32) or microbiological failure (*p* = 0.076).

**Conclusions:**

Oral step‐down therapy for Gram‐negative BSIs appears to be a valuable alternative to prolonged IV treatment, potentially reducing IV exposure, complications and healthcare costs without compromising clinical or microbiological outcomes.

**Trial Registration:**

PROSPERO registration number: CRD420251056335

## Graphical abstract

Forest plots of mortality, clinical failure and microbiological failure in patients receiving oral stepdown or continuing intravenous treatment for Gram‐negative bloodstream infections.

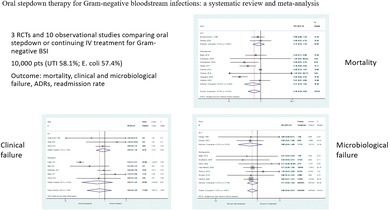

## 1 Introduction

In recent years, the management of Gram‐negative bloodstream infections (GN‐BSIs) has significantly changed [1]. Traditionally, prolonged intravenous antibiotic courses have been used to ensure adequate drug exposure and to reduce the risk of relapse. Increasing attention, however, has shifted towards early oral therapy as a strategy of reducing complications associated with extended hospitalization, including nosocomial infections and higher healthcare costs [2].

Oral therapy offers several potential advantages, such as early discharge of patients and prevention of catheter‐related adverse events. Drugs with high oral bioavailability, such as fluoroquinolones, trimethoprim‐sulfamethoxazole or selected oral beta‐lactams, can achieve adequate therapeutic concentrations for the treatment of GN‐BSIs. Evidence from observational studies and randomized controlled trials indicates that early switch to oral therapy may be as effective as continued intravenous treatment with respect to mortality and infection recurrence [3, 4, 5]. Nevertheless, important uncertainties remain. Beta‐lactams have been traditionally considered drugs with low bioavailability, unable to achieve adequate concentrations to treat severe or deep‐seated infections when administered orally. At the same time, several concerns have been raised in recent years regarding the tolerability and the spread of resistance to fluoroquinolones, which represent one of the most commonly used oral options for the treatment of Gram‐negative infections. Moreover, the safety of early switch and its impact on microbiological clearance are still a matter of debate.

In this systematic review and meta‐analysis we aimed at comparing clinical and microbiological outcomes of patients receiving oral step‐down therapy or continuing intravenous treatment for GN‐BSIs in order to synthesize available evidence on effectiveness and safety of oral switch strategies in this setting.

## 2 Methods

### 2.1 Search Strategy and Selection Criteria

We conducted a systematic review and meta‐analysis of observational studies and randomized controlled trials (RCTs) with the aim of comparing mortality rate, microbiological failure, clinical failure, rate of Adverse Drug Reactions (ADRs) and rate of hospital readmission of patients with bloodstream infections (BSI) due to Gram‐negative bacteria receiving oral stepdown within 5 days from the start of antibiotic therapy or continuing intravenous antimicrobial treatment for the entire course of therapy. The study was carried out in accordance with the indication of PRISMA guidelines [6]. The protocol was registered on PROSPERO database.

The PICO question used was as follows:

*Population*: patients with BSI due to Gram negative bacteria.

*Intervention*: oral step‐down within 5 days from the start of antimicrobial therapy.

*Comparator*: continuation of intravenous antibiotic treatment for the entire duration of the therapy.

*Outcome*: mortality rate, microbiological failure, clinical failure, grade 3–4 ADRs, hospital readmission rate.

*Study design*: experimental or observational studies (case–control, cohort, case series).

Items used in the search and other data on methods are shown in Methods S1.

We used the following inclusion criteria: (a) present original data from experimental and observational studies; (b) compare clinical and microbiological outcomes of patients who switched within 5 days to an active oral antibiotic (oral penicillin or oral cephalosporins or fluoroquinolones or cotrimoxazole) or continued an intravenous antimicrobial for the treatment of a Gram negative BSI; (c) report at least one of the following outcomes: overall mortality, clinical failure rate, microbiological failure rate, rate of ADRs and rate of hospital readmission; (d) include at least 10 patients; (e) be published as a full paper from inception to April 2025; (f) be written in English language.

Conversely, papers were excluded if they were: (a) meta‐analysis, letters, reviews, meeting abstracts or editorial comments; (b) duplicate publications.

Three researchers (I.D.L., M.F., G.R.) independently evaluated the title and abstracts of all citations to evaluate them for inclusion, reporting for each study the reasons of exclusion. Thus, the selected articles were retrieved as full tests to assess their eligibility. In case of disagreement an additional researcher (L.O.) checked all the papers and took the definite decision.

### 2.2 Data Analysis

The quality appraisal of each study was conducted by two reviewers (I.D.L., L.O.) independently using the Cochrane Risk of Bias Tool version 2 (RoB2) [7] for RCT, and the Newcastle Ottawa Scale [8] for observational studies. In case of discrepancy a third reviewer (N.C.) decided.

As primary outcome we evaluated all‐cause mortality either at 30‐day or 90‐day. Pre‐planned secondary endpoints included clinical failure, microbiological failure, rate of ADRs and rate of hospital readmission. Risk ratios (RRs) were the meta‐analytic measure used. A further sub‐analysis was performed including only studies whose results were controlled for confounding factors with the propensity score. Finally, for the primary outcome we conducted sub‐analyses including only patients with urinary tract infections, patients with BSI due to Enterobacterales, as well as studies in which patients had a median time to switch to oral treatment of ≤ 3 days or > 3 days.

Heterogeneity among studies was assessed using the *I* ^2^. *I* ^2^ values up to 49% were considered as low heterogeneity, while values between 50% and 75% and 75% denoted moderate and high heterogeneity, respectively; a *p* value < 0.1 for Q statistics was considered statistically significant. All analyses were conducted with a random‐effects model. Publication bias was evaluated through funnel plot inspection, and Egger's test was applied only when ≥ 10 studies were included [9]; in this case, a *p* value < 0.10 was deemed significant. Unless otherwise specified, tests were two‐sided, and significance was set at *p* < 0.05. Statistical analyses were performed using Stata/IC version 16 software (StataCorp, College Station, TX, USA).

Since a systematic review and meta‐analysis the informed consent of patients and the approval by ethical committee were not needed.

## 3 Results

### 3.1 Characteristics of Studies Enrolled

**FIGURE 1:**
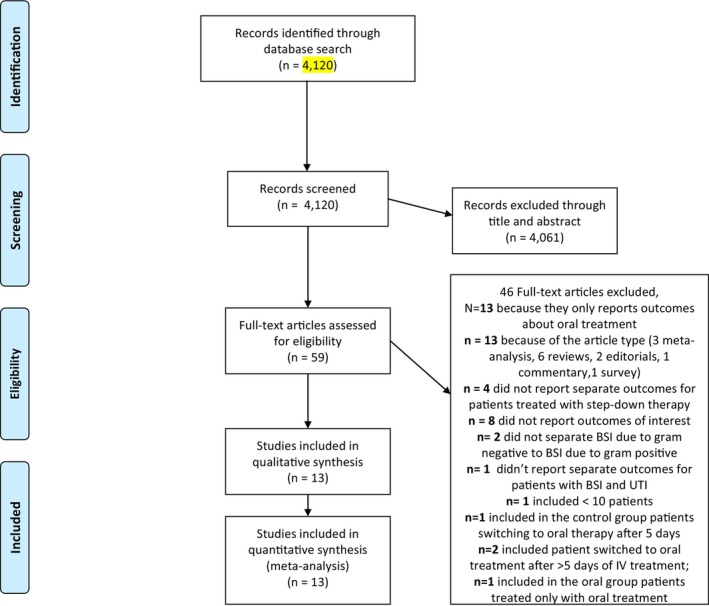
PRISMA flow diagram of the process of identification and selection of articles included in the meta‐analysis.

The flow chart of the article's selection is presented in Figure 1. We started screening 4120 original papers and then we excluded 4061 of them after reading the title and the abstract. After evaluating as full text, of the remaining 59 papers we excluded 46 additional studies because they did not meet the inclusion criteria. Hence, a total of 13 studies [3, 4, 5, 10, 11, 12, 13, 14, 15, 16, 17, 18, 19] were included in the analysis, accounting for 10,000 patients enrolled.

We summarized the main characteristics of the selected studies in Table 1; we took into account 3 randomized controlled trials (RCTs) [4, 10, 11] and 10 retrospective cohort studies [3, 5, 12, 13, 14, 15, 16, 17, 18, 19]. The samples size ranged from 50 to 4581 patients; 8 of the studies [3, 5, 10, 13, 14, 16, 17, 19] were conducted in the US, 3 in Europe (Spain [12], Belgium [11] and Denmark [18]) and 2 in Asia (1 in Thailand [15] while one was a multinational study including patients from Bahrain, Kuwait, Qatar and Türkiye [4]).

**TABLE 1:** Baseline characteristics of the 13 studies included in the meta‐analysis.

First author, year (References)	No. of patients	Enrolment period	Country	Study design	Age, median (IQR)	Males (*n*, %)	Source of bloodstream infection/severity (*n*, %)	Pathogens isolated/resistance profile (*n*, %)	Patients receiving oral step‐down (*n*, %)	Duration (days, median, IQR)	Antimicrobials used for oral step‐down (*n*, %)	Patients treated with IV treatment only (*n*, %)	IV drugs used (*n*, %)	Duration (days, median, IQR)	Time to oral switch (days, median, IQR)
Amodio‐Groton, 1996 [10]	50	October 1987–August 1989	USA	RCT	62.1 (IQR 28–91)	20 (40)	UTI 35(70) Biliary tract 3 (6) Other 7 (14)	*E. coli* 32 (64) *K. pneumoniae* 7 (14) Other Enterobacterales 12 (24) Non‐fermenting 3 (6) Other 3 (6)	24 (48)	9.8 (6.1) (mean, SD)	Ciprofloxacin 24 (100)	26 (52)	Mezlocillin or ampicillin + gentamicin 11 (22) Cephalosporin ± gentamicin 13 (26) TMP/SMX 4 (8) Other 9 (18)	15.7 (±9.7)	3
Engers, 2024 [5]	4581	January 1–December 31 2019	USA	Retrospective cohort study	67 (IQR 55–77)	2389 (52.2%)	UTI 2285 (49.9) Biliary tract 528 (11.5) CVC‐related 315 (6.9) Other 1453 (31.7)	*E. coli* 2362 (51.6) Klebsiella spp. 916 (20) Other Enterobacterales 764 (16.7) Non‐fermenting 504 (11) ESBL 328 (7.16)	1969 (43)	11 (IQR 9–14)	FQs 1224 (62.2) TMP/SMX 227 (11.5) BL/BLI 150 (7.6)‐ Cephalosporins 408 (20.7) Other 17 (0.9) Combination 47 (1)	2612 (57)	NR	13 (IQR 8–16)	5 (IQR 4–6)
Gangji, 1989 [11]	65	NR	Belgium	RCT	NR	35 (53.8)	UTI 33 (50.8) Biliary tract 6 (9.2) Other 11 (16.9) Unknown 15 (23.1) Severe infection 32 (49.2)	*Enterobacterales* 65 (100)	29 (44.6)	13.8 (mean)	Ciprofloxacin 29 (100)	36 (55.4)	Ciprofloxacin 36 (100)	Mean 11.8	3
Meije, 2019 [12]	101	January 2004–December 2015	Spain	Retrospective cohort study	NR	62 (61.4)	UTI 65 (64) Biliary tract 15 (14) Catheter‐related 6 (6) Other 8 (8) Unknown 7 (7) Sepsis/septic shock 22 (21.8)	*E. coli* 80 (79.2) *K. pneumoniae* 21 (20.8) ESBL or AmpC 101 (100)	37 (36.7)	12 (IQR 10–14)	TMP/SMX 25 (67.6) FQs 9 (24.3) BL/BLI 1 (2.7) Other 2 (5.4)	59 (58.4)	Carbapenems 59 (100)	12 (IQR7.17)	2.5 (IQR 0–6)
Nussbaum, 2024 [13]	162	January 2016–December 2021	USA	Retrospective cohortl study	NR	84 (51.9)	UTI 104 (64.2) Biliary tract 15 (9.3) Other 22 (13.5) Unknown 21 (12.9)	*E. coli* 76 (45.7) Klebsiella spp. 38 (23.5) Other Enterobacterales 29 (17.9) Non‐fermenting 9 (5.6) Other 6 (3.7) Polymicrobial 4 (2.5)	120 (74.1)	15 (IQR 7–36)	FQs 104 (86.7) TMP/SMX 5 (4.2) BL/BLI 6 (5) Cephalosporins 3 (2.5) Other 2 (1.7)	42 (25.9)	Cephalosporins 31 (73.8) Carbapenems 11 (26.2)	16 (IQR 12–29)	4 (IQR 1–15)
Olson, 2022 [14]	174	January 2002–January 2020	USA	Retrospective cohort study	NR	96 (55.2)	UTI 174 (100)	*E. coli* 160 (91.9) Klebsiella spp. 3 (1.8) Other Enterobacterales 10 (5.7) Non‐fermenting Gram negative 1 (0.6)	73 (42)		BL/BLI 9 (12.3) TMP/SMX 7 (9.6) Ciprofloxacin 3 (4.1) Cephalosporins 43 (58.9) Other 13 (17.8)	101 (58)	Ampicillin 3 (3) Cephalosporins 71 (70.3) Carbapenems 2 (2) Other 4 (4)		≤ 4
Omrani, 2023 [4]	174	October 2009–March 2022	Bahrain, Kuwait, Qatar, Turkiye	RCT	56.6 (16.7)	89 (51%)	UTI 105 (60.3) Biliary tract 12 (6.9) Other 57 (32.8) Septic shock 5 (2.8)	*E. coli* 115 (66.1) Klebsiella spp. 42 (24.1) Other Enterobacterales 17 (9.8) ESBL 28 (16.1)	89 (51.1)	14 (IQR 11–16)	BL/BLI 27 (30.3) Cephalosporins 31 (34.8) FQs 17 (19.1) TMP/SMX 14 (15.7)	85 (48.9)	BL/BLI 15 (17.6) Carbapenems 23 (27) Cephalosporins 44 (51.7) FQs 2 (2.3) TMP/SMX 1 (1.2)	11 (IQR 8–14)	3–5
Pradubkham, 2022 [15]	955	August 2015–July 2020	Thailand	Retrospective cohort study	71 (IQR 60–81)	408 (42.7)	UTI 371 (38.8) Primary BSI 211 (22.1) Other 373 (39.1) Septic shock 258 (27)	*E. coli* 594 (59.1) *K. pneumoniae* 159 (7.9) Other Enterobacterales 94 (9.8) Non‐fermenting Gram negative bacteria 92 (9.2) Other 65 (6.8) MDR 309 (32.3)	545 (57.1)		FQs 306 (56.1) TMP/SMX 10 (1.8) BL/BLI 56 (10.3) Cephalosporins 168 (30.8) Others 5 (0.9)	410 (42.9)	Cephalosporins 206 (50) Carbapenems 140 (34) FQs 9 (2.2) BL/BLI 56 (13.7)		3 (IQR 1–4)
Rieger, 2017 [16]	241	July 2010–June 2015	USA	Retrospective cohort study	64 (IQR 54–74)	113 (47)	UTI 241 (100)	*E. coli* 137 (56.8) *K. pneumoniae* 55 (22.8) Polymicrobial 2 (1) MDR 72 (29.9)	135 (56)	14 (IQR 14–14)	FQs 88 (65.3) Beta‐lactams 26 (19) TMP/SMX 12 (9.1)	106 (44)	Cephalosporins 84 (79.2) Carbapenems 6 (5.8)	14 (IQR 13–14)	4 (IQR 2–5)
Tamma, 2019 [20]	1478	January 2008–December 2014	USA	Retrospective cohort study	59 (IQR 48–69)	769 (52)	Pneumonia 58 (3.9) UTI 594 (40.2) Biliary tract 210 (14.2) Catheter‐associated 272 (18.4) Other 337 (22.9)	*E. coli* 645 (43.6) *K. pneumoniae* spp. 505 (34.2) Other Enterobacterales 328 (22.2)	739 (50)	14 (IQR 11–16)	FQs 518 (59.1) TMP/SMX 99 (13.4) BL/BLI 38 (5.1) Cephalosporins74 (9.6)	739 (50)	NR	14 (IQR 11–16)	3 (IQR 2–4)
Thurber, 2018 [17]	346	January 2008–June 2016	USA	Retrospective cohort study	NR	150 (43.4)	UTI 346 (100)	*E. coli* 229 (66.2) Klebsiella spp. 49 (13.3) Other Enterobacterales 39 (11.3) Non‐fermenting Gtam Negative 29 (8.4)	264 (76.3)	14 (IQR 13–15)	FQs 229 (87) TMP/SMX 21 (8) BL/BLI 6 (2) Cephalosporins 7 (2.6) Other 1 (0.4)	82 (23.7)	FQs 5 (69) Carbapenems 24 (29) Cephalosporins 49 (59.8) BL/BLI 2 (2) Other 2 (2)	15 (IQR 14–15.5)	3 (IQR 2–4)
Tingsgård, 2024 [18]	914	January 2018–December 2021	Denmark	Retrospective cohort with target trial emulation study	74.5 (IQR 63.38–83.2)	512 (56)	UTI 699 (76.5) Gastrointestinal infection 156 (17.1) Other 59 (6.5)	*E. coli* 693 (75.8) Klebsiella spp. 114 (12.5) Other Enterobacterales 58 (6.3) Other 49 (5.4) ESBL 10 (1.1)	433 (47.4)	NR	Ciprofloxacin 152 (16.6) TMP/SMX 4 (1) Others NR	481 (52.6)	NR	NR	4
Veillette, 2023 [19]	759	January 2016–December 2022	USA	Retrospective cohort study	NR	289 (38.1)	UTI 759 (100) Septic shock 97 (33.5)	*E. coli* 622 (81.9) Klebsiella spp. 137 (18.1) ESBL 28 (3.7)	651	13	FQs 289 (44.4) TMP/SMX 73 (11.2) Beta‐lactams 289 (44.4)	108	Beta‐lactams 108 (100)	15	0.7–1.2

Abbreviations: BL/BLI, beta‐lactam/beta‐lactamase inhibitor; CVC, central venous catheter; ESBL, extended‐spectrum beta‐lactamase; FQs, fluoroquinolones; IQR, interquartile range; IV, intravenous; MDR, multidrug‐resistant; NR, not reported; RCT, randomized controlled trial; TMP/SMX, trimethoprim‐sulfamethoxazole; UTI, urinary tract infections.

Among the 10,000 patients included, 5016 (50.2%) received oral step‐down; in particular 2992 patients (59.6%) were switched to fluoroquinolones, 487 (9.7%) to cotrimoxazole and 1335 (26.6%) to an oral beta‐lactam; the median time to switch ranged from 0.7 to 5 days. Eight studies reported clear patients' eligibility criteria for oral switch [3, 4, 11, 13, 15, 16, 17, 18], including ability to take oral medication [3, 4, 11, 15, 16], hemodynamic stability [3, 4, 11, 17, 18], and achievement of adequate source control by day 3–5 [3, 4, 13, 15, 18]. The median duration of treatment ranged from 9.8 to 15 days in the oral stepdown group and from 11 to 16 days in the intravenous treatment group.

The principal clinical syndrome associated with BSI was urinary tract infection (UTI) in 5811 patients (58.1%), followed by hepatobiliary infections (789, 7.9%) and central line associated bloodstream infection (CLABSI) 593 (5.9%). The prevalence of patients with septic shock ranged from 2.8% to 33.5%. Regarding the isolates, the most isolated pathogens included were *E. coli* in 5745 (57.4%) cases and *Klebsiella* spp. in 2046 (20.5%). The prevalence of Enterobacterales resistant to third generation cephalosporins ranged from 1.1 to 100%.

About the outcomes assessed, 5 studies evaluated 30‐day mortality [3, 12, 13, 15, 16], while three studies reported 90‐day mortality [4, 18, 19] and one paper 21‐day mortality [17]; one study did not report any information about timing evaluating mortality [10].

Nine studies described clinical failure: for 6 papers it was reported as a composite outcome including all‐cause mortality, relapse of BSI and transition back from oral to IV medication or requiring new antibiotic therapy or infection‐related hospital re‐admission [4, 12, 15, 16, 17, 19]; for 2 studies it was considered as lack of signs and symptoms' resolution or recurrence of clinical syndrome [5, 10] and 1 study did not report any precise definition [11]. The microbiological failure was defined as persistence or recurrence of the BSI at 30‐day in 6 papers [3, 12, 13, 14, 15, 19], 21‐day [17] and 90‐day [4] in one paper each. To estimate rate of ADRs we included 6 studies: 2 studies considered as ADRs *Clostridioides difficile* infection [13, 19], 2 studies IV‐line associated complications [13, 17], and the remaining any unfavourable and unintended sign, symptoms or disease associated with the use of the study treatments [4, 10, 11]. Finally, to estimate the rate of hospital readmission, three studies described this rate at 30 days [5, 14, 16] and two at 90 days from discharge [4, 19].

### 3.2 Quality Appraisal of Studies Included in the Analysis

The quality appraisal of the included papers is shown in Tables S1 and S2. Considering the three RCTs included in our meta‐analysis, two showed some concerns regarding risk of bias [4, 10], while one was judged to have a high risk of bias [11].

Among the 10 cohort studies, only two were assessed as having an intermediate risk of bias according to the Newcastle–Ottawa Scale [13, 17], whereas eight out of 10 were judged to have a low risk of bias [3, 5, 12, 14, 15, 16, 18, 19].

### 3.3 Meta‐Analysis of Studies Evaluating the Mortality of Patients Receiving Oral Stepdown or Continuing IV Treatment for the Entire Course of Therapy

**FIGURE 2:**
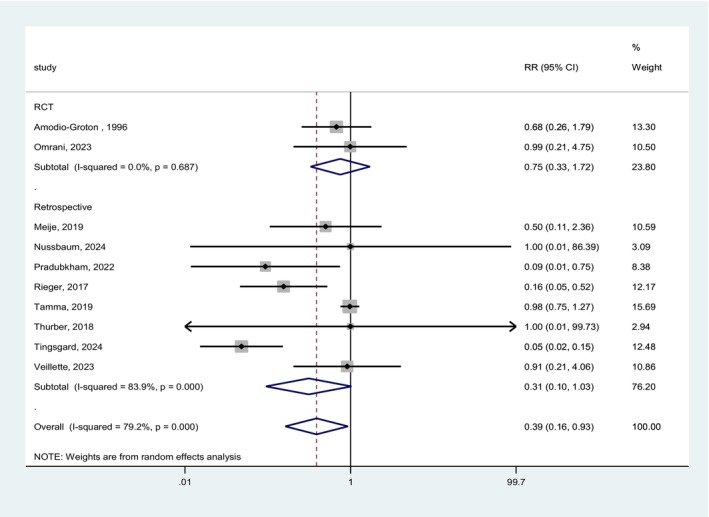
Forest plot of RRs of mortality in patients switched to oral treatment or continuing intravenous treatment.

The results of the analysis of all the outcomes analysed are displayed in Table 2. In the analysis comparing all‐cause mortality of patients that received oral step‐down or continuing IV antibiotic treatment, we included 10 papers [3, 4, 10, 12, 13, 15, 16, 17, 18, 19] involving a total of 5176 patients (Table 2): 125 patients died in the oral step‐down group and 210 died in the IV group. The meta‐analysis demonstrated significant lower mortality among patients switching to oral treatment (RR: 0.39, 95% CI: 0.16–0.93; *p* = 0.034) (Table 2, Figure 2). Nevertheless, significant heterogeneity was observed among studies (*I* ^2^ = 79.2%, *p* < 0.001) and some concerns regarding the potential presence of publication bias emerged from the visual inspection of the funnel plot (Figure S2) and through the Egger's test (*p* = 0.017). Though, when we performed a sub‐analysis including propensity score‐adjusted data, evaluating 5 studies [3, 12, 13, 15, 19] and 3459 patients, of which 2097 received oral step‐down (Table 2, Figure S1), we found no difference in mortality rate among treatment groups (RR: 0.71, 95%, CI: 0.36–1.40; *p* = 0.32). A low heterogeneity was demonstrated among studies (*I* ^2^ = 28.2%, *p* = 0.234). When evaluating only patients with BSI form urinary source, no difference in mortality emerged among the 1411 subjects switched to oral treatment and the 625 who continued intravenous therapy enrolled in 4 studies [16, 17, 18, 19] (RR: 0.54, 95%, CI: 0.20–1.42; *p* = 0.211, Table 2, Figure S1). The same results were obtained in the sub‐analysis including 2507 patients with BSI due to *Enterobacterales* enrolled in 4 studies [3, 4, 12, 19], a similar mortality rate was observed in the two groups of patients (RR: 0.96, 95%, CI: 0.75–1.23; *p* = 0.75), with no statistical heterogeneity detected. Finally, no significant difference among groups emerged in the analysis including the 6 studies [3, 10, 12, 15, 17, 19] (3693 patients) with a median time to switch to oral treatment ≤ 3 days (RR: 0.79, 95%, CI: 0.51–1.23; *p* = 0.29), while in the 4 studies for a total of 1483 patients with a median time to switch > 3 days [4, 13, 16, 18] patients in the oral group showed a lower mortality rate, almost reaching the statistical significance (RR: 0.521 95%, CI: 0.04–1.00; *p* = 0.051) (Table 2, Figure S1).

**TABLE 2:** Meta‐analyses of studies comparing mortality, clinical failure, microbiological failure, rate of ADRs and hospital readmission rate among patients switched to oral treatment or those continuing intravenous treatment.

Outcome	No. of studies	No. of patients receiving oral step‐down/continuing IV therapy	No. of events	RR	95% CI	*p*	Heterogeneity test (*I* ^2^, %; *p*)
Mortality	10	3037/2139	125 (4.1)/210 (9.8)	0.39	0.16–0.93	**0.034**	79.2; < 0.001
Mortality (PS‐adjusted)	5	2097/1362	111 (5.3)/115 (8.4)	0.71	0.36–1.40	0.32	28.2; 0.234
Mortality (patients with UTI)	4	1411/625	40 (2.8)/46 (7.3)	0.54	0.20–1.42	0.211	55.2;0.082
Mortality (patients with *Enterobacterales* BSI)	4	1515/992	113 (7.4)/110 (11.1)	0.96	0.75–1.23	0.75	0;0.87
Mortality (time to oral switch ≤ 3 days)	6	2265/1428	116 (5.1)/123 (8.6)	0.79	0.51–1.23	0.29	15.7; 0.313
Mortality (time to oral switch > 3 days)	4	772/711	9 (1.2)/87 (9.4)	0.21	0.04–1.00	0.051	72.4;0.012
Clinical failure	8	3091/3413	117 (3.8)/486 (14.2)	0.65	0.26–1.65	0.369	90.2; < 0.001
Microbiological failure	9	2546/1659	95 (3.7)/56 (3.4)	0.69	0.49–0.97	**0.032**	0; 0.891
Microbiological failure (PS‐adjusted)	6	2170/1459	84 (3.9)/42 (2.9)	0.71	0.49–1.04	0.076	0; 0.628
ADRs	5	2911/3008	474 (16.3)/596 (19.8)	0.84	0.61–1.15	0.281	51.1; 0.085
Hospital readmission	5	2917/3011	478 (16.4)/599 (19.9)	0.85	0.62–1.16	0.301	52.7;0.076

*Note:* *p* values < 0.05 are displayed in bold.

Abbreviations: ADRs, adverse drug reactions; BSI, bloodstream infection; CI, confidence interval; IV, Intravenous; PS, propensity score; RR, relative risk; UTI, urinary tract infection.

### 3.4 Meta‐Analysis of Studies Evaluating the Clinical Failure of Patients Receiving Oral Stepdown or Continuing IV Treatment for the Entire Course of Therapy

**FIGURE 3:**
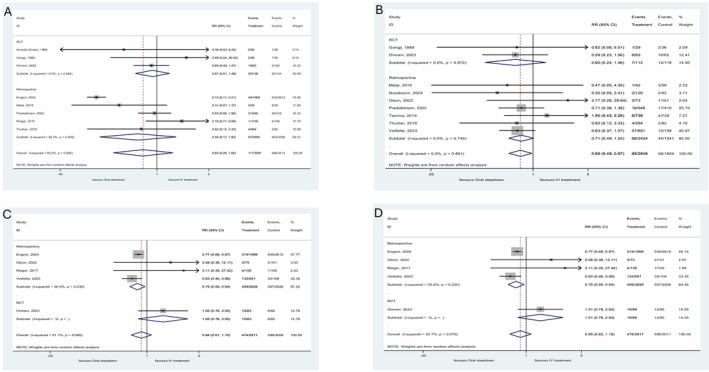
Forest plot of RRs of clinical failure (A), microbiological failure (B), ADRs (C) and hospital readmission (D) in patients switched to oral treatment or continuing intravenous treatment.

Eight studies [4, 5, 10, 11, 12, 15, 16, 17] were included for the analysis of the clinical failure: lower, but not significantly, clinical failure rate was observed in the 3091 patients switched orally compared to the 3413 treated entirely IV (RR: 0.65, 95% CI: 0.26–1.65; *p* = 0.369); even in this case a high heterogeneity was observed (*I* ^2^ = 90.2%, *p* < 0.001) (Table 2, Figure 3A). Not enough studies reported propensity score‐adjusted data to perform a sub‐analysis.

### 3.5 Meta‐Analysis of Studies Evaluating the Microbiological Failure of Patients Receiving Oral Stepdown or Continuing IV Treatment for the Entire Course of Therapy

Nine studies evaluated the microbiological failure [3, 4, 11, 12, 13, 14, 15, 17, 19], including 95 subjects with microbiological failure out of 2546 in the oral group versus 56 out of 1659 in IV group: we observed a lower rate in the oral step‐down group (RR = 0.69, 95% CI: 0.49–0.97; *p* = 0.032) with no heterogeneity detected between studies (*I* ^2^ = 0%, *p* = 0.891) (Table 2, Figure 3B).

Similar results, though not reaching the statistical significance, were obtained when we analysed the 6 studies with propensity score‐adjustment [3, 12, 13, 14, 15, 19] comparing 2170 versus 1459 patients (RR: 0.71, 95% CI: 0.49–1.04; *p* = 0.076), with no heterogeneity among studies (*I* ^2^ = 0%, *p* = 0.628) (Table 2, Figure S1b).

### 3.6 Meta‐Analysis of Studies Evaluating the Rate of ADRs of Patients Receiving Oral Stepdown or Continuing IV Treatment for the Entire Course of Therapy

Five studies compared the rate of ADRs [4, 5, 14, 16, 19] for a total of 5919 subjects, and no significant differences between the two groups of treatment (RR: 0.85, 95% CI: 0.62–1.16; *p* = 0.301) with a moderate heterogeneity among studies (*I* ^2^ = 52.7%, *p* = 0.076) (Table 2, Figure 3C). Not enough data were available for the sub‐analysis including propensity score‐adjusted studies.

### 3.7 Meta‐Analysis of Studies Evaluating the Rate of Hospital Readmission of Patients Receiving Oral Stepdown or Continuing IV Treatment for the Entire Course of Therapy

Finally, when we compared the hospital readmission rates between the 2917 patients in the oral and the 3011 patients in the IV group included in 5 studies [4, 5, 14, 16, 19], no difference was observed between the 2 groups (RR: 0.84, 95% CI: 0.61–1.15; *p* = 0.281) with a moderate heterogeneity emerged among studies (*I* ^2^ = 51.1%, *p* = 0.085) (Table 2, Figure 3D).

No evidence of publication bias for the secondary end‐points emerged from the visual inspection of funnel plots (Figure S3).

## 4 Discussion

In the present systematic review and meta‐analysis, aimed to describe the clinical and microbiological outcomes of patients with BSI due to GN bacteria treated with oral step‐down therapy compared to patients continuing IV antibiotics for the entire course of therapy, we included 13 studies enrolling 10,000 subjects with GN‐BSI, mainly from urinary source. A total of 5015 subjects (50.2%) received oral antibiotics as step‐down therapy within 5 days from the start of treatment. In the present meta‐analysis, a lower rate of all‐cause mortality and microbiological failure was observed in the oral step‐down group, while no significant differences in clinical failure emerged among the two groups. Since most of the studies had a retrospective design, we conducted a sub‐analysis focusing on data adjusted for propensity score to reduce confounding bias, which demonstrated no significant differences in mortality nor in microbiological failure rates between the two groups.

In recent years, the transition from IV to oral therapy has gained increasing importance in the management of bacterial infections and continues to represent a topic of great clinical interest, particularly in the management of BSI due to Gram‐negative bacteria. Traditionally, the management of GN‐BSI has relied on prolonged IV antibiotic treatment to ensure adequate systemic exposure and favourable pharmacokinetic properties. However, although clinically effective, this approach is associated with substantial logistical, economic and safety drawbacks, including prolonged hospitalization, increased healthcare costs and nosocomial infections, such as catheter‐related complications.

There is a growing body of evidence suggesting that, in clinically stable patients with GN‐BSI, predominantly from a urinary source, early transition to oral antibiotics represents a feasible therapeutic strategy. For example, in a multicenter cohort of 1478 patients with BSI due to *Enterobacterales*, Tamma et al. [3] reported no excess mortality in patients transitioning to oral step‐down therapy, provided patients were clinically stable and the source of infection was controlled.

Our results are consistent with previous meta‐analyses evaluating the early transitioning from IV to oral treatment strategies in bloodstream infections and sepsis. In the recent meta‐analysis by Li and colleagues [21], including 38 studies and 11,566 subjects with bacteremia and/or sepsis due to Gram positive or Gram negative strains, the primary outcome, that is, treatment failure, in patients transitioning to oral antibiotics resulted non‐inferior to those who continued IV treatment, even in the subgroup analysis focusing on uncomplicated bacteremia (*n* = 1290; OR 0.66; 95% CI 0.41–1.09; *I* ^2^ = 47.7%) and BSI due to *Enterobacterales* (*n* = 508; OR 0.59; 95% CI 0.32–1.08; *I* ^2^ = 8.1%). Moreover, no significant differences between the two groups were found in overall 90‐day mortality, relapse and readmission rates; subgroup analysis on *Enterobacterales* were not available on these topics. Our meta‐analysis confirmed similar results, that is, in the subgroup of patients with *Enterobacterales* BSI a comparable mortality rate among treatment groups. This sub‐analysis included a significantly higher number of patients (2507 as compared to 508 of the above mentioned meta‐analysis). As well, Mohammed and colleagues [22] in a previous meta‐analysis including 8 observational studies and 1 RCT for a total of 9782 patients with Gram‐negative BSI, demonstrated a higher treatment success rate (OR 1.42, 95% CI 1.00–2.01, *p* = 0.05), a lower risk of all‐cause mortality (OR 0.22, 95% CI 0.07–0.67, *p* = 0.008) and a shorter in‐hospital stays (OR −4.85, 95% CI −7.02 to −2.69, *p* < 0.001) in patients with GN‐BSI transitioning to oral therapy within the first week of admission. Moreover, patients who switched to oral treatment did not show a difference in hospital readmission rate compared to patients who continued IV therapy in both subgroup analysis (in GN‐BSI OR 0.81, 95% CI 0.36–1.84, *p* = 0.62). However, no propensity‐score adjustment was conducted in this analysis, so a clear comparability among groups cannot be demonstrated. Similarly, we observed a lower mortality and microbiological failure rate in the oral treatment group in the unadjusted analysis; however, these differences were not demonstrated in the propensity‐score adjusted analysis, that was not present in previous meta‐analyses. This discrepancy could be explained by considering that patients with less severe disease and easier to treat pathogens are more likely to be switched to oral treatment in clinical practice; when evaluating patients with comparable clinical and microbiological characteristics through propensity‐score adjustment, the difference previously reported in mortality and microbiological failure among groups were not confirmed, although a trend towards better outcomes in the oral treatment group remained. Nevertheless, most available evidence regards patients with uncomplicated infections, mostly from urinary source and due to susceptible pathogens, with an early clinical response to intravenous therapy, and thus may not be generalizable to more complex infections or situations.

Another key point emerged from our analysis, that was not addressed in the previous papers [21, 22] is the timing of switch to oral treatment. Firstly, we decided to exclude from our analysis studies reporting a time to oral stepdown > 5 days, since several data have demonstrated that a total treatment of 7 days is noninferior to longer duration in the management of uncomplicated Gram‐negative bloodstream infections [23, 24, 25]. Moreover, we conducted a sub‐analysis including only studies that reported a median time to switch < 3 days, and no significant difference in term of overall mortality was observed among groups, suggesting that a very early switch in clinically stable patients is an effective option.

Two international guidelines on the management of Gram‐negative infections recommend transitioning from IV to oral therapy in any bacterial infections, whenever possible, that is, in patients who are clinically stable, who are able to take oral medication, when an effective oral option is available and no concerns about intestinal absorption are present, and source control has been achieved [20, 26]. This recommendation takes into consideration both stewardship aspects, including a reduced risk of nosocomial infections, avoidance of central or midline catheters placement, and shorter hospital length of stay, and, for the first time, explicitly patient‐centered factors, such as preferences for home‐based treatment and the perceived ease of taking oral medications [26]. The strongest evidence in favour of oral step‐down comes from urinary‐source GN‐BSI, whereas data for/from non‐urinary sources, deep‐seated infections or immunocompromised hosts [18, 27] remain sparse. Indeed, Tingsgård et al. [18] noted that outcomes are less certain in these settings, and Meije et al. [12] and Thurber et al. [17] caution against extrapolating oral step‐down benefits to these higher‐risk populations, where prolonged IV therapy may still be warranted.

Despite encouraging findings, literature remains dominated by several methodological weaknesses. Most studies are retrospective, with high heterogeneity in inclusion criteria, oral regimens and follow‐up periods, and MICs are inconsistently reported. Follow‐up is typically limited to 30 days, potentially missing late recurrences. Selection bias is unavoidable, as patients transitioned to oral therapy were typically younger, clinically stable and infected with susceptible organisms. Moreover, nowadays the therapeutic drug monitoring (TDM) will represent a valuable tool to help clinicians to maintain oral antibiotics concentrations within a targeted therapeutic range.

Regarding the main limitations of our meta‐analysis, all about three studies have a retrospective design, and in most cases, there was not a clear comparability in terms of severity of disease, patients' comorbidities and relevant microbiological characteristics. This could have introduced a significant risk of selection bias and limits the reliability of the results, considering that in clinical practice stable patients with less severe disease are more likely to be switched to oral therapy. Not enough data was available to conduct sub‐analyses according to the clinical severity of infections or the Charlson Comorbidity Index of patients. However, the propensity‐score matched analysis may have partially overcome this limitation. No papers differentiated outcomes based on pathogens isolated or resistance profile, and no sub‐analysis considering the primary site of BSI or disease severity could be performed. Also, due to considerable heterogeneity in the antibiotics administered—both oral and IV—it was not feasible to perform a sub‐analysis comparing outcomes associated with different antibiotic agents. Furthermore, different dosing for oral agents has been administered among studies, and this could have significantly impacted the clinical and microbiological outcomes, particularly for less bioavailable drugs, such as beta‐lactams.

Finally, some of the analysis showed a high heterogeneity, therefore, it was necessary to conduct a sub‐analysis including only studies adjusted for propensity score, which reduced the sample size.

On the other hand, the good quality of most studies, the significant number of patients included and the availability of meaningful clinical and microbiological outcomes as well as of propensity‐score matched results strengthen the findings of our analysis.

## 5 Conclusions

In conclusion, our study confirms that oral step‐down appears to be a valuable alternative to full IV therapy in GN‐BSI with similar clinical and microbiological outcomes, including all‐cause mortality. Further prospective data or clinical trials are needed to confirm which is the best time to switch to oral therapy, and to better characterize the microbiological profile, patients' profile and clinical syndrome that suits an early transitioning to oral or an entirely oral treatment.

## Author Contributions

L.O. and N.C. designed the study; L.O., N.C., I.D.L. and C.M. drafted the manuscript and were involved in the interpretation of the data and critical revision of the manuscript, I.D.L., M.F. and G.R. screened the citations and assessed the eligibility of the articles; I.D.L. and L.O. extracted the data and performed the quality assessment; L.O. ran the statistical analysis.

## Funding

This work was supported in part by PRIN: PROGETTI DI RICERCA DI RILEVANTE INTERESSE NAZIONALE—Bando 2022 PNRR, Ministero dell'Università, project ‘Analysis of resistome and virulome among carbapenem‐resistant Gram‐negative bacteria and their correlation with clinical and microbiological outcomes in a cohort of hospitalized patients with colonization or infection: a multicentre prospective study’.

## Conflicts of Interest

The authors declare no conflicts of interest.

## Supporting information


**Data S1:** eci70244‐sup‐0001‐DataS1.docx.

**Methods S1:** eci70244‐sup‐0002‐DataS2.docx.

**Table S1:** eci70244‐sup‐0003‐TableS1‐S2.docx.

**Figure S1:** eci70244‐sup‐0004‐FigureS1‐S3.pptx.

## Data Availability

The data that support the findings of this study are available from the corresponding author upon reasonable request.
