# Supplementary material for: Oral Stepdown Therapy for Gram‐Negative Bloodstream Infections: A Systematic Review and Meta‐Analysis
Source: Eur J Clin Invest. 2026 Aug 13;56(8):e70244. doi: 10.1111/eci.70244 (PMC13472257; doi:10.1111/eci.70244)
Supplement: Supplementary file 1 [file ECI-56-e70244-s001.docx]

**ORAL STEPDOWN THERAPY FOR GRAM-NEGATIVE BLOODSTREAM INFECTIONS: A SYSTEMATIC REVIEW AND META-ANALYSIS**

Lorenzo Onorato MD, PhD^1^, Ilaria De Luca MD^1^, Marika Ferrante MD^1^, Giuseppe Ricciardiello MD^1^, Caterina Monari MD^1^, Nicola Coppola MD, PhD^1^

1. Department of Mental Health and Public Medicine, Section of Infectious Diseases, University of Campania Luigi Vanvitelli, Via L. Armanni 5, 80131, Naples, Italy.

**METHODS S1**

Three authors (IDL, MF, GR) searched two of the most comprehensive databases, MEDLINE and EMBASE, for original reports published up to April 2025; for the electronic research were used the following ~~items~~ string: (“Gram negative” OR “Enterobacterales” OR “Pseudomonas”) AND (“BSI” OR “bloodstream infection” OR “bacteremia”) AND (“oral therapy” OR “oral treatment” OR “fluroquinolones” OR “cotrimoxazole” OR “oral penicillins”, OR “oral cephalosporins”). Also, a manual search among references listed in all papers that we retrieved as full text was conducted to identify additional eligible studies. The Cochrane Library was not searched because the access was not available at our institution.

Three researchers (IDL, MF, GR) independently evaluated the title and abstracts of all citations to evaluate them for inclusion, reporting for each study the reasons of exclusion. Thus, the selected articles were retrieved as full tests to assess their eligibility. In case of disagreement an additional researcher (LO) checked all the papers and took the definite decision.
