# Supplementary material for: Oral Stepdown Therapy for Gram‐Negative Bloodstream Infections: A Systematic Review and Meta‐Analysis
Source: Eur J Clin Invest. 2026 Aug 13;56(8):e70244. doi: 10.1111/eci.70244 (PMC13472257; doi:10.1111/eci.70244)
Supplement: Supplementary file 2 [file ECI-56-e70244-s002.docx]

|  | **First author, year** | **Amodio-Groton, 1996** | **Gangji, 1989** | **Omrani, 2023** |
| --- | --- | --- | --- | --- |
| **Domain 1: Randomization process** | **1.1 Random sequence generation** | Y | Y | Y |
|  | **1.2 Allocation concealment** | NI | NI | NI |
|  | **1.3 Baseline imbalance** | N | N | N |
|  | **Risk of bias judgement** | Some concerns | Some concerns | Some concerns |
| **Domain 2: Deviations from intended interventions** | **2.1 Patients’ awareness about intervention** | PY | PY | PY |
|  | **2.2 Carers’ awareness about intervention** | Y | Y | Y |
|  | **2.3 Deviations from intended intervention because of trial context** | PN | PN | PN |
|  | **2.4 Deviations likely to affect the outcome** | - | - | - |
|  | **2.5 Deviations balanced between groups** | - | - | - |
|  | **2.6 Appropriateness of analysis** | Y | N | Y |
|  | **2.7 Impact of failure to analyse participants in the group to which they were randomized** | - | PY | - |
|  | **Risk of bias judgement** | Some | High | Some |
| **Domain 3: Missing outcome data** | **3.1 Data available for all participants** | Y | N | Y |
|  | **3.2 Evidence that the results were not biased** | - | PN | - |
|  | **3.3 Could missingness depend on its true value?** | - | PY | - |
|  | **3.4 Is it likely missingness depend on its true value?** | - | PY | - |
|  | **Risk of bias judgement** | Low | High | Low |
| **Domain 4: Measurement of the outcome** | **4.1 Methods of outcomes measurement inappropriate** | N | N | N |
|  | **4.2 Different methods among groups** | N | N | N |
|  | **4.3 Outcomes assessors awareness of intervention** | NI | PY | PY |
|  | **4.4 Outcome assessment influenced by knowledge on intervention** | N | N | N |
|  | **4.5 Outcome assessment likely to be influenced by knowledge** | - | - | - |
|  | **Risk of bias judgement** | Low | Low | Low |
| **Domain 5: Selection of the reported results** | **5.1 Pre-specified analysis plan** | NI | NI | NI |
|  | **5.2 Results selected from multiple outcome measures** | N | N | N |
|  | **5.3 Results selected from multiple analysis of the data** | N | N | N |
|  | **Risk of bias judgement** | Some | Some | Some |
| **Overall judgement** | **Risk of bias judgement** | Some concerns | High risk of bias | Some concerns |

**Supplementary Table 1**. Quality assessment of RCTs included in the meta-analysis

| **First author, year,** | **Selection** | | | | **Comparability** | **Outcomes** | | | **Total score** |
| --- | --- | --- | --- | --- | --- | --- | --- | --- | --- |
|  | **Representativeness**  **of the exposed**  **cohort** | **Selection of**  **the nonexposed**  **cohort** | **Ascertainment**  **of**  **exposure** | **Outcome of interest**  **not present at**  **start of study** | **Comparability**  **based on design**  **or analysis^^1^** | **Assessment**  **of**  **outcome** | **Was follow-up**  **long enough for**  **outcomes to occur?^^^2^** | **Adequacy**  **of follow-up**  **of cohorts^3^** |  |
| **Engers, 2024** | * | * | * | * | ** | * | * |  | 8* |
| **Meije, 2019** | * | * | * | * | ** | * | * | * | 9* |
| **Nussbaum, 2024** |  |  | * | * | ** | * | * |  | 6* |
| **Olson, 2022** |  |  | * | * | ** | * | * | * | 7* |
| **Pradubkham, 2022** | * | * | * | * | ** | * | * | * | 9* |
| **Rieger, 2017** | * | * | * | * | * | * | * |  | 7* |
| **Tamma, 2019** | * | * | * | * | ** | * | * | * | 9* |
| **Thurber, 2018** | * | * | * | * | * | * |  |  | 6* |
| **Tingsgard, 2024** | * | * | * | * | ** | * | * | * | 9* |
| **Veillette, 2023** | * | * | * | * | ** | * | * | * | 9* |

**Supplementary Table 2**: Quality assessment of cohort studies included in the meta-analysis

^1^ * sex, age; ** severity defined as SOFA, PITT, ICU admission

^2^ At least 28 days

^3^ Less than 20% of the patients included lost at follow up
